# Current evidence of generative artificial intelligence specifically developed for dental and maxillofacial radiology: a systematic review

**DOI:** 10.1093/dmfr/twag024

**Published:** 2026-05-21

**Authors:** Kuo Feng Hung, Feng Wang, Ming-Yi Lu, Xieqi Shi, Michael M Bornstein, Qi Yong H Ai

**Affiliations:** Oral and Maxillofacial Radiology, Applied Oral Sciences and Community Dental Care, Faculty of Dentistry, The University of Hong Kong, Hong Kong SAR, 0000, China; Oral and Maxillofacial Surgery, Faculty of Dentistry, The University of Hong Kong, Hong Kong SAR, 0000, China; School of Dentistry, College of Oral Medicine, Chung Shan Medical University, Taichung, 402306, Taiwan; Department of Stomatology, Chung Shan Medical University Hospital, Taichung, 402306, Taiwan; Section of Oral Maxillofacial Radiology, Department of Clinical Dentistry, University of Bergen, 7804, Norway; Department of Oral Health & Medicine, University Center for Dental Medicine Basel UZB, University of Basel, Basel, 4058, Switzerland; Department of Imaging and Interventional Radiology, Faculty of Medicine, The Chinese University of Hong Kong, Prince of Wales Hospital, Hong Kong SAR, 0000, China

**Keywords:** generative artificial intelligence, dento-maxillofacial radiology, image enhancement, synthetic image generation, automated interpretation

## Abstract

**Objectives:**

This systematic review aimed to investigate the current development and application landscape of generative artificial intelligence (Gen-AI) networks specifically designed for dental and maxillofacial radiology (DMFR) and summarize their potential applications for clinical practice, education, and research.

**Methods:**

Five electronic databases were searched to identify studies that developed and validated DMFR-specific Gen-AI networks. Data regarding the purpose and type of the Gen-AI model, dataset details, quantitative evaluation metrics, methods of subjective assessment, and key findings were extracted. Customized assessment criteria adapted from the Checklist for Artificial Intelligence in Medical Imaging (CLAIM) checklist were used to evaluate the risk-of-bias for the included studies in 4 domains (dataset details, reporting of Gen-AI model architecture and training strategies, reliability of performance evaluation methods, and accessibility of code and developed models).

**Results:**

Forty-three studies were included from the initially identified 2060 records. Of these, 24 studies (55.8%) focused on image quality, addressing improvements in spatial resolution, artifact and noise reduction, image geometry and projection, quantitative accuracy of voxel values, 14 (32.6%) on image simulation (including the generation of bitewing, panoramic, and cephalometric radiographs, image-to-image translation, and post-treatment prediction simulation), 3 (7%) on 3D reconstruction from 2D images, and 2 (4.6%) on automated interpretation and reporting. Nearly all included studies (95.3%) reported objective evaluation metrics while about half (58.1%) incorporated subjective assessments using scoring systems or visual grading. Risk-of-bias was moderate for dataset details in 7 studies (16.3%) and performance evaluation in 20 (46.5%), and high for code and model accessibility in 35 studies (81.4%).

**Conclusions:**

While DMFR-specific Gen-AI models show promising potential for clinical practice, education, and research, their applicability requires overcoming challenges related to data quality, validation, integration, and ethical and legal considerations. Further clinical validation, increased transparency and accessibility, and thorough evaluation of cost-effectiveness in diagnostic workflows are essential to ensure their safe and effective use.

## Introduction

Generative artificial intelligence (Gen-AI) represents a transformative and rapidly evolving branch of AI that enables machines not only to analyze or interpret data, but also to create entirely new content.^1^ By using advanced algorithms and deep learning architectures, Gen-AI models are capable of generating original text, images, audio, video, and other data types through the identification and replication of complex patterns within large-scale datasets. This generative capability distinguishes Gen-AI from conventional AI systems (eg, machine learning classifiers and deep learning computer vision models), which are mainly designed for tasks such as recognizing patterns, making predictions, classifying or categorizing data, detecting anomalies, identifying objects in images, segmenting images, extracting features, and analyzing or interpreting existing information.^2–5^

Large language models (LLMs) and large vision-language models (LVLMs) are advanced types of models within Gen-AI.^6^ General-purpose LLMs (such as GPT-3 and GPT-4) and LVLMs (such as GPT-4V) are trained on broad and diverse datasets to perform a wide range of tasks, rather than specializing in any particular field. Applying these general-purpose models to tasks that require a high level of domain expertise can lead to less reliable outputs.^7^ In contrast, many specialized, domain-specific Gen-AI models were fine-tuned on tailored datasets to achieve expert-level performance in targeted areas, such as BioGPT for biomedical text mining or Med-PaLM for answering medical questions.^8^

Medical imaging has emerged as a major application area for Gen-AI within healthcare.^9^ In radiology, Gen-AI models are increasingly being developed to synthesize images, improve image quality, support automated analysis, and expand datasets for rare conditions for AI training.^10^ These models have the potential to improve diagnostic accuracy and workflow efficiency. Innovations in Gen-AI are now extending into sub-specialties including dental and maxillofacial radiology (DMFR).^11^ While previous reviews have addressed AI-driven computer vision models and general LLMs in DMFR,^6^^,^^12^^,^^13^ the development and application of DMFR-specific Gen-AI networks have not been systematically investigated. Therefore, this study aimed to systematically review the current development and application landscape of DMFR-specific Gen-AI networks and summarize their potential applications for clinical practice, education, and research.

## Methods

This systematic review was reported in accordance with the Preferred Reporting Items for Systematic Reviews and Meta-Analyses (PRISMA) guidelines.^14^ The protocol for this study was registered with the International Prospective Register of Systematic Reviews (PROSPERO) under Registration ID: CRD420261284251. The focused question used for the literature search was “What are current development and application of Gen-AI networks specifically designed for DMFR?” Studies were considered eligible if they addressed this question. In this systematic review, the PICO framework was used to define the eligibility criteria and guide the literature search. The Population (P) comprised studies involving DMFR images, including intraoral, panoramic, cephalometric, cone-beam CT (CBCT), CT, and MRI of the oral and maxillofacial region. The Intervention (I) was the application of Gen-AI models designed for DMFR-specific tasks. The Comparison (C) involved conventional imaging methods, original (non-AI-enhanced) images, or other standard or AI-based approaches. The Outcomes (O) were the objective and subjective performance metrics of the Gen-AI models. The PICO elements are summarized in Table 1.

**Table 1:** Description of the “P (population) I (intervention) C (comparator) O (outcomes)” elements used in framing the research question and the search strategy.

Criteria	Specification
Focus question	“What are current development and application of generative artificial intelligence (Gen-AI) networks specifically designed for dental and maxillofacial radiology (DMFR)?”
Population	Studies involving DMFR images, including intraoral, panoramic, cephalometric, CBCT, CT, and MRI, of the oral and maxillofacial region
Intervention	Application of Gen-AI models designed for DMFR-specific tasks
Comparator	Conventional imaging methods, original (non-AI-enhanced) images, or other standard or AI-based approaches
Outcome	Objective and subjective performance metrics of the Gen-AI models
Search strategy	Group 1 (Generative AI model terms, combined with ORs): terms relating to generative artificial intelligence models Group 2 (Oral/Dental/Maxillofacial imaging terms, combined with ORs): terms identifying oral, dental, and maxillofacial radiology and imaging AND: The 2 groups are combined with AND, so only articles containing at least one term from each group are retrieved.

Abbreviations: CBCT = cone-beam CT; DMFR = dental and maxillofacial radiology; Gen-AI = generative artificial intelligence.

### Search strategy and selection process

A comprehensive search was carried out in December 2025 across 5 electronic databases, including PubMed, Medline (Ovid), Embase (Ovid), Web of Science, and Scopus. The search approach used 2 primary concept groups, each containing relevant synonyms and related terms, connected within the group using the Boolean operator OR. The first group included terms associated with Gen-AI, while the second focused on DMFR-related terms. The 2 groups were then combined using the Boolean operator AND to retrieve articles that contain at least one term from both groups. The search terms and syntax were adapted for each individual database. Complete search strategies for all databases are provided in Table S1. All records were imported into EndNote Version 21 (Clarivate Analytics, Philadelphia, United States), where titles were automatically screened for duplicates. No restrictions on publication period were applied during the database search.

The study selection process was carried out in 2 phases. Initially, 2 experienced reviewers (K.F.H. and Q.Y.H.A.) independently screened the titles and abstracts of all retrieved records. Records were selected for full-text review if the title and abstract suggested potential relevance to both Gen-AI and DMFR. Records were excluded if they met any of the following exclusion criteria: (1) involved Gen-AI applications unrelated to DMFR tasks, (2) were not focused on DMFR-specific Gen-AI, (3) were not original research articles (such as reviews, letters, or editorials), (4) were conference proceedings, (5) were not published in English, and (6) were inaccessible in full-text. If the relevance of a record could not be determined based on its title and abstract, it was included for full-text assessment.

The same reviewers independently evaluated the full-text of the selected records to determine their final eligibility. Studies were included if they met the following criteria:

1. Studies focusing on DMFR-specific Gen-AI, involving images or text data related to anatomical structures and/or pathological findings in the oral and maxillofacial regions;
2. Studies using clinical or publicly available imaging datasets containing any of the following image modalities: intraoral, panoramic, cephalometric, CBCT, CT, and MRI for the development, training, or evaluation of Gen-AI models; and
3. Studies reporting objective quantitative evaluation metrics and/or subjective assessment outcomes.

Furthermore, the reference lists of the included original studies were screened to identify additional eligible studies. Any disagreements between reviewers were resolved through discussion and consensus.

### Data extraction

The same 2 reviewers (K.F.H. and Q.Y.H.A.) independently carried out data extraction using a standardized template in an Excel spreadsheet (Microsoft Corporation, Redmond, Washington, United States). The extracted data included the author, publication year, objective of the proposed Gen-AI network, type of Gen-AI model, dataset details, quantitative evaluation metrics, methods of subjective assessment, and key findings. Any uncertainties encountered during the data extraction process were resolved through consensus.

### Risk of bias assessment

The risk of bias in the included studies was evaluated using customized assessment criteria (Table 2) adapted from the updated Checklist for Artificial Intelligence in Medical Imaging (CLAIM),^15^ focusing on 4 main domains (dataset details, reporting of Gen-AI model architecture and training strategies, reliability of performance evaluation methods, and accessibility of code and developed models). These domains were selected to address critical questions affecting study reliability: (1) Was the source and nature (paired or unpaired) of the dataset clearly described? (2) Were the Gen-AI model architecture and training strategies comprehensively reported? (3) Was the reliability of model performance evaluation demonstrated through both objective (quantitative metrics) and subjective (expert or clinician-based) assessments? (4) Were the codes or developed models for the Gen-AI systems openly accessible?

**Table 2:** Customized assessment criteria, adapted from the updated CLAIM,^15^ used to evaluate the risk of bias in the included studies with a specific focus on 4 major domains of bias.

Risk of bias			
Dataset details	Reporting of Gen-AI model architecture and training strategies	Reliability of performance evaluation methods	Accessibility of code and developed models
Clearly describe the source of the images and/or text used in the study Specify whether paired or unpaired data were used for training, testing, or both training and testing	Clearly describe the Gen-AI network architecture (model type, components, modifications) Clearly describe training strategies (preprocessing, loss functions, optimization, augmentation)	Clearly describe how the Gen-AI network’s performance was evaluated in comparison to other networks, human observers, or established standards Specify the objective quantitative metrics used for evaluation Specify the subjective qualitative methods (eg, expert/clinician-based evaluation of the generated outcomes) used	Provide valid access to the code or models for the proposed Gen-AI systems to ensure they are openly accessible

Abbreviation: Gen-AI = generative artificial intelligence.

For dataset details, studies were assessed on (1) whether they clearly described the source of images and/or text, and (2) whether they specified if paired or unpaired data were used for training, testing, or both. For reporting of Gen-AI model architecture and training strategies, studies were evaluated on whether they clearly described (1) the network architecture (including model type, components, and modifications) and (2) the training strategies (such as preprocessing, loss functions, optimization, and augmentation). For reliability of performance evaluation methods, studies were assessed based on whether they provided (1) a clear description of how the network’s performance was evaluated (including comparisons to other networks, human observers, or standards), specified (2) objective quantitative metrics (eg, structural similarity index measure [SSIM], root mean squared error [RMSE], mean squared error [MSE]), and/or (3) subjective qualitative methods (eg, expert or clinician-based evaluation). For accessibility of code and developed models, studies were assessed on whether valid access to the code or models was provided.

Each domain was rated on a 3-point scale (low, moderate, or high risk of bias). A “low” risk-of-bias rating was given when all required items in a domain were reported; “moderate” when any single required item was missing; and “high” when none of the required items were reported. The risk of bias assessment was performed independently by 2 reviewers (K.F.H. and Q.Y.H.A.) who were provided with a detailed description of the rating system. No formal calibration was conducted prior to the assessment. Any disagreements in scoring were resolved through discussion to reach consensus.

## Results

**Figure 1:**
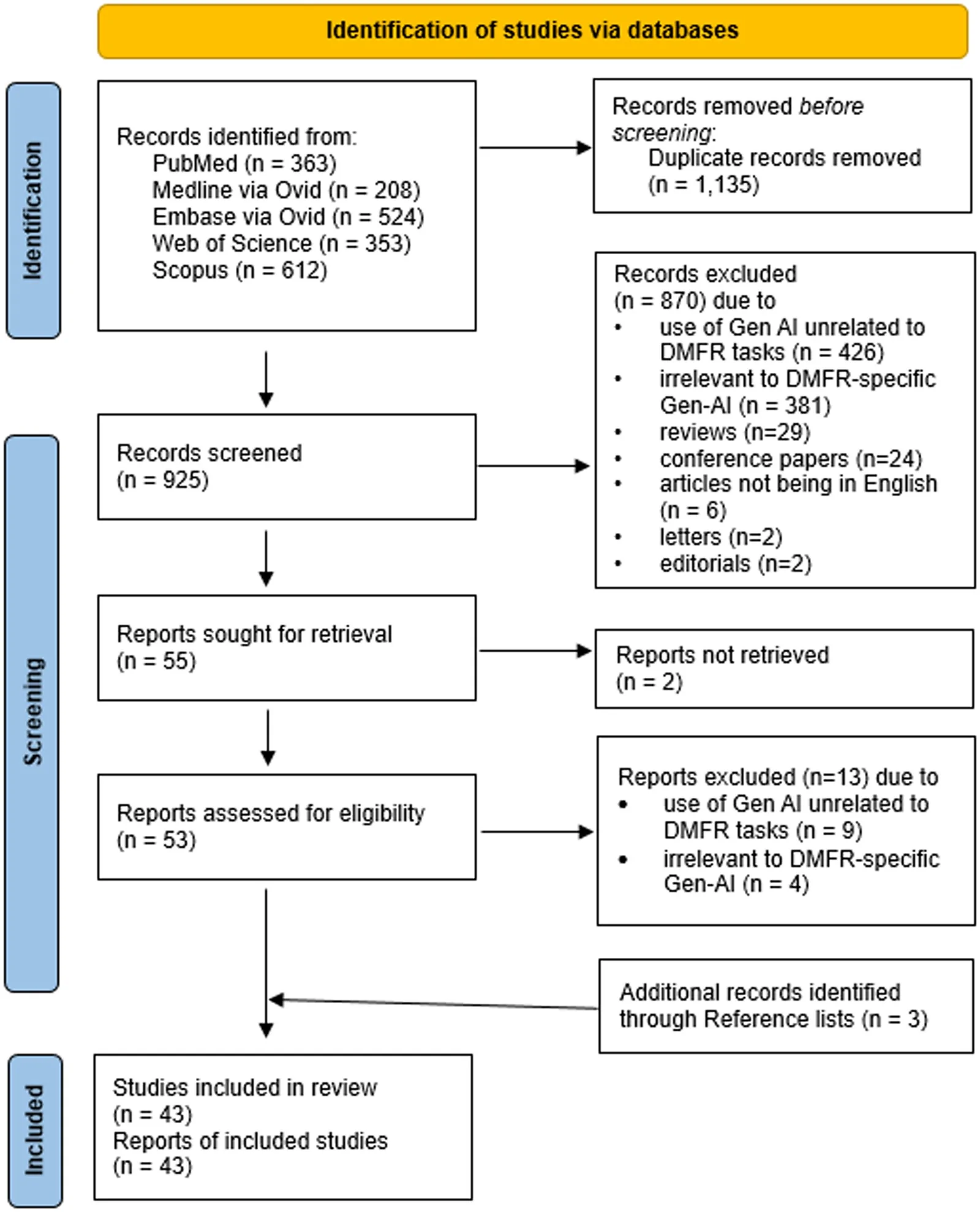
PRISMA flowchart illustrating the study selection process. Abbreviations: DMFR = dental and maxillofacial radiology; Gen-AI = generative artificial intelligence.

A total of 2060 records were identified through the search. After removing 1135 duplicates, 925 records remained for title and abstract screening. Subsequently, 55 articles were selected for full-text assessment. Fifteen articles were excluded due to use of Gen-AI unrelated to DMFR tasks (*n* = 9), irrelevance to DMFR-specific Gen-AI (*n* = 4), and full-text not available (*n* = 2) (Table S2). Three additional eligible studies were identified through reference list screening. Therefore, 43 original studies were included in this systematic review. The selection process is illustrated in Figure 1.

**Figure 2:**
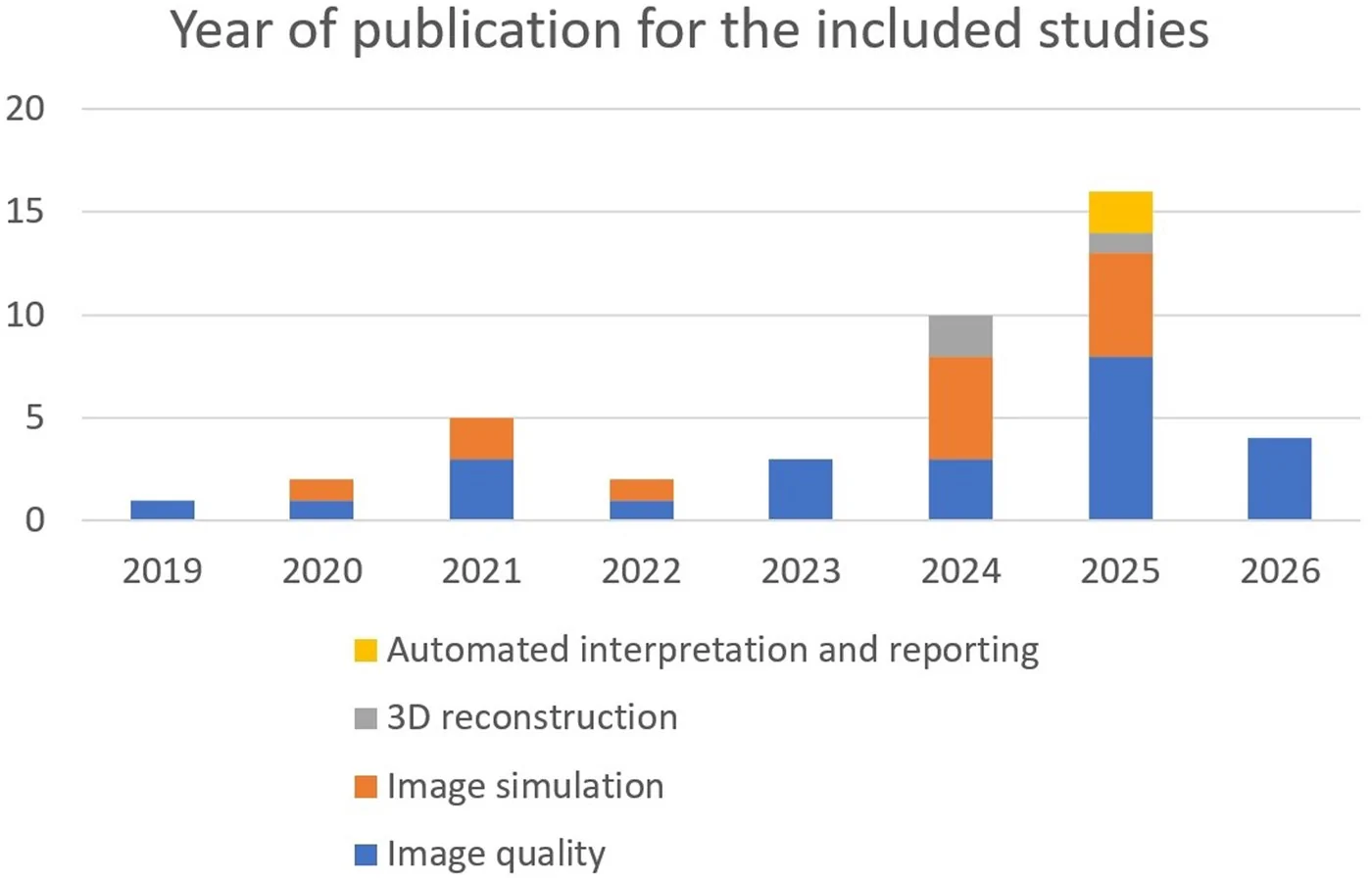
The number of studies published each year from 2019 to 2026, categorized by 4 main categories: image quality enhancement (blue), image simulation (orange), 3D reconstruction (gray), and automated interpretation and reporting (yellow).

Of the 43 included studies published between 2019 and 2026, 70% (30 out of 43) were published since 2024 (Figure 2). These studies developed and validated DMFR-specific Gen-AI networks for a range of imaging tasks. They were grouped into 4 main categories based on the model’s intended task, and each category was further subdivided according to the underlying methodological approach. The categories included (1) image quality, addressing improvements in spatial resolution,^16–21^ image geometry and projection,^22–24^ noise reduction,^25^^,^^26^ quantitative accuracy of voxel values,^27^^,^^28^ and artifact reduction^29–39^; (2) image simulation, involving the generation of bitewing, panoramic, and cephalometric radiographs,^40–47^ image-to-image translation,^48–51^ and post-treatment prediction simulation^52^^,^^53^; (3) 3D reconstruction, including reconstruction with incomplete resources and from 2D images^54–56^; and (4) automated interpretation and reporting^57^^,^^58^ (Tables 3 and 4).

**Table 3:** Purposes of DMFR-specific Gen-AI models in the included studies.

Category	Sub-category	Function of the DMFR-specific Gen-AI models
Image quality (*n* = 24)	Spatial-resolution (*n* = 6)	Enhance the spatial resolution of periapical radiographs Enhance the spatial resolution of panoramic radiographs Enhance the spatial resolution of CBCT images
	Improvement of image geometry and projection (*n* = 3)	Reduce blur in panoramic radiographs Correct distortions in panoramic radiographs Enhance image quality of CBCT Standardize style across panoramic radiographs from different devices
	Noise reduction (*n* = 2)	Denoise low-dose CBCT
	Quantitative accuracy of voxel values (*n* = 2)	Improve the accuracy of Hounsfield unit measurements in CBCT Quantify bone mineral density from CBCT
	Artifact reduction (*n* = 11)	Remove ring artifacts from CBCT Reduce metal artifacts in CBCT Reduce exomass-related metal artifacts in CBCT Remove artifacts from high-density materials in CBCT Reduce motion artifacts in CT and CBCT Reduce shading artifacts in CBCT
Image simulation (*n* = 14)	Simulation without corresponding resources (*n* = 8)	Generate bitewings Generate panoramic radiographs Generate cephalometric radiographs Generate task-specific panoramic radiographs for diagnostic purposes, such as those depicting jaw cysts
	Image-to-image translation (*n* = 4)	Generated synthetic cephalometric radiographs from low-dose CBCT projections Synthetize CT from CBCT images Synthetize high-quality micro-CT images from CBCT Synthetize contrast-enhanced CT from non-contrast CT Synthetize T2-weighted MRI from proton density-weighted MRI for TMJ disc segmentation
	Post-treatment prediction simulation (*n* = 2)	Generate reconstructed 3D mandibular morphology from CBCT Generate predicted 3D post-orthodontic facial changes from CBCT
3D reconstruction (*n* = 3)	Reconstruction with incomplete resources (*n* = 1)	Reconstruct 3D CBCT from incomplete projections
	Reconstruction from 2D images (*n* = 2)	Reconstruct 3D images of impacted canines from 2D panoramic radiographs Reconstruct 3D volumetric model of the oral cavity from 2D panoramic radiographs
Automated interpretation and reporting (*n* = 2)	Disease-specific multimodal diagnostic and treatment support system (*n* = 1)	Diagnose osteonecrosis of the jaw from panoramic radiographs, segment lesion areas, recommend treatment plans, provide automated radiology reports, and support clinicians through interactive Q&A
	Dental radiology report generation and multilingual patient Q&A system (*n* = 1)	Generate detailed radiology reports, detect major dental diseases, and interact with patients via a Q&A system

Abbreviations: CBCT = cone-beam CT; DMFR = dental and maxillofacial radiology; Gen-AI = generative artificial intelligence.

**Table 4:** Overview of the included studies on Gen-AI applications in DMFR.

Author	Year	Main purpose of Gen-AI network	Type of Gen-AI network	Dataset	Quantitative evaluation metrics	Subjective assessment method	Key results	Conclusion
**Image quality (spatial-resolution)**								
Moran et al.^16^	2021	To enhance the spatial resolution of periapical radiographs	SRGANs	228 periapical radiographs from Policlínica Piquet Carneiro, Rio de Janeiro and 120 images from Universiti Teknologi Malaysia	MSE, PSNR, and SSIM	Mean opinion score rated by a dentist	*Objective* SRGAN with/without transfer learning MSE: 13.19-14.79/16.99 PSNR: 37-37.7/36.4 SSIM: 1/1 *Subjective* SRGAN with/without transfer learning Score: 3.4/3.6	The use of SRGANs with transfer learning significantly improved the resolution and quality of periapical radiographs over existing interpolation and deep learning methods.
Mohammad-Rahimi et al.^17^	2023	To enhance the spatial resolution of panoramic radiographs	SRCNN, SRGAN, U-Net, SwinIR, LTE	888 panoramic radiographs collected from a single center in Tehran, Iran	MSE, PSNR, and SSIM	Mean opinion score by 4 dentists using a 5-point scale	*Objective* LTE achieved the best results including MSE: 7.42, PSNR: 39.74, and SSIM: 0.92. SRCNN was close behind LTE in most metrics. *Subjective* LTE received the highest score (3.59), close to the score for original high-resolution images (3.97).	LTE and SRCNN significantly enhanced the visual quality and resolution of panoramic radiographs.
Celik et al.^18^	2024	To enhance the spatial resolution of panoramic radiographs	SRCNN, ESPCNN, SRGAN, and Autoencoder	1714 panoramic radiographs from 3 open datasets	SSIM and PSNR	NA	SSIM ranged from 0.82 to 0.98; PSNR from 28.7 to 40.2 across all models and scaling factors. SRCNN achieved the best overall performance (highest SSIM and PSNR)	SRCNN substantially improved the quality and detail of dental panoramic radiographs.
Chen et al.^19^	2024	To synthesize high-quality micro-CT images from CBCT scans	Variation-driven neural network	50 pairs of CBCT and micro-CT images of extracted individual teeth	SSIM, PSNR, NMSE, DICE, and ASSD	NA	The method achieved superior results over traditional GAN-based approaches and other baselines in generating micro-CT images from CBCT (SSIM: 0.92, PSNR: 23.45, and NMSE: 0.25); the model achieved improved accuracy in segmenting pulp and crown (DICE: 0.82, and ASSD: 4.65)	The proposed network enables the generation of high-quality micro-CT images from CBCT scans.
Chen et al.^20^	2026	To improve the spatial resolution of CBCT images using micro-CT as reference images	Enhanced super-resolution generative adversarial network (ESRGAN) and hybrid attention transformer (HAT)	48 pairs of CBCT and micro-CT images of extracted individual teeth	PSNR and SSIM 3D reconstruction of the root canal system and overlap analysis with micro-CT references	Unclear number of endodontists evaluated images using a 5-point scale without clear criteria for scoring	*Objective* The proposed models with edge loss function achieved the highest PSNR (28.46; HAT) and highest SSIM (0.72; ESRGAN). 3D reconstructions showed ESRGAN with edge loss function had greatest overlap with micro-CT ground truth. *Subjective* No statistically significant difference between the ratings of the proposed models with edge loss function and those of micro-CT	Micro-CT-guided super-resolution with edge-aware optimization substantially improves the diagnostic utility of CBCT in endodontics.
Sabry et al.^21^	2025	To enhance the quality of panoramic radiographs and automated oral disease detection	DentoSMART-LDM, the framework combining metaheuristic optimization and a pathology-aware latent diffusion model	82 300 panoramic radiographs containing 12 dental and jaw conditions	SSIM, PSNR, FID, LPIPS, diagnostic accuracy, F1, AUC	12 oral pathologists and 8 oral radiologists rated overall image quality, diagnostic feature clarity, clinical usefulness using a 5-point scale and provided diagnostic interpretation and confidence	*Objective* SSIM: 0.94, PSNR: 34.82, FID: 6.73, LPIPS: 0.012, AUC: 0.99, overall accuracy: 97.3%, F1 Score: 0.97 *Subjective* Overall image quality: 4.73/5 (3.18 for traditional enhancement, 2.47 for no enhancement) Diagnostic confidence: 4.68/5 Clinical usefulness: 4.69/5 Diagnostic accuracy improvement: +17.4% Expert confidence gain: +23.4%	DentoSMART-LDM enhanced the quality of panoramic radiographs and improved diagnostic accuracy and confidence.
**Image quality (improvement of image geometry and projection)**								
Kim et al.^22^	2023	To enhance the quality of panoramic radiographs, especially in cases of image degradation due to blur and noise	Pix2Pix GAN model (U-Net-based generator, Patch-GAN discriminator)	400 panoramic radiographs from Yonsei University Dental Hospital, including 100 images processed to simulate 4 types of distortions: blurriness, noise, blur + noise, anterior blur	PSNR and SSIM	Two oral radiologists scored image quality using a clinical evaluation chart (max score: 46)	*Objective* Highest improvement in images with blur in the anterior teeth (PSNR: 36.27, SSIM: 0.90). Lowest improvement in images with blur and noise (PSNR: 32.74, SSIM: 0.82). *Subjective* Mean quality score for original images: 44.6/46 Enhanced images from the blur group scored higher than originals (45.2). Enhanced images from the noise group had the lowest score (36).	The Pix2Pix GAN model can quantitatively and qualitatively enhance degraded panoramic radiographs, especially those degraded by blur.
Kolukisa et al.^23^	2025	To restore and enhance the diagnostic quality of panoramic radiographs affected by blurriness, noise, and anterior-region-specific blurriness	Pix2Pix, CycleGAN, and RegGAN	400 panoramic radiographs from Yonsei University Dental Hospital, including 100 images processed to simulate 4 types of distortions: blurriness, noise, blur + noise, anterior blur	PSNR and SSIM	Two oral radiologists scored image quality using a clinical evaluation chart	*Objective* Pix2Pix achieved the highest PSNR and SSIM across all distortion types, CycleGAN showed moderate improvements, and RegGAN showed negligible improvements *Subjective* Radiologists’ ratings were highest for Pix2Pix-restored images, followed by CycleGAN and RegGAN.	Pix2Pix is the most effective GAN model for restoring panoramic radiographs across a range of common distortions compared to CycleGAN and RegGAN.
Kim et al.^24^	2025	To harmonize panoramic radiographs from different equipment	CycleGAN	15 624 unpaired panoramic images from 2 devices (R-unit and P-unit) and 222 pairs of images from the same patients	FID and LPIPS	Two oral radiologists classified transformed images as R-unit, P-unit, or undetermined	*Objective* Transformed P-unit images had lower FID (7.36 vs. 8.38) and LPIPS (0.49 vs. 0.52) compared to original P-unit images, both indicating greater similarity to R-unit images. *Subjective* Of 60 transformed P-unit images, 43.3-46.7% were classified as R-unit, 20.0%-28.3% as P-unit, and 28.3%-33.3% as undetermined.	CycleGAN can harmonize the style of panoramic radiographs from different equipment, making images more uniform across devices.
**Image quality (noise reduction)**								
Hegazy et al.^25^	2020	To denoise low-dose CBCT	Wasserstein GAN with U-net generator (U-WGAN)	3006 pairs of high-dose and low-dose CBCT image slices from 2 human skull phantoms	PSNR and SSIM	NA	PSNR: 32.75 (U-net), 27.42 (U-WGAN), 23.96 (WGAN) SSIM: 0.974 (U-net), 0.962 (U-WGAN), 0.953 (WGAN)	Transfer learning using U-WGAN is effective for CBCT image denoising.
Zhang et al.^26^	2026	To enhance the visibility of the mandibular canal (MC) and periodontal ligament (PL) of mandibular third molars (M3Ms) on low-dose CBCT	Pix2Pix, CycleGAN, and diffusion models	151 pairs of standard- and low-dose CBCT scans	SSIM, PSNR, MAE, and RMSE	3 clinicians (GP, OMFS, oral radiologist) evaluated anatomical visibility (MC, PL), M3M-MC proximity, root morphology, adjacent molar status, surgical approach, and referral decisions.	*Objective* Gen-AI-enhanced low-dose CBCT images showed significantly improved quality (higher PSNR, lower MAE and RMSE). *Subjective* AI-enhanced images significantly improved MC visibility for all clinicians and PL visibility for GP and OMFS; no significant differences between standard-dose, original low-dose, and AI-enhanced low-dose images in risk assessment, treatment strategies, or referral decisions; observers could not reliably distinguish AI-enhanced images from originals (correct identification rates of 49.7%-58.8%).	Gen-AI-enhanced low-dose CBCT images significantly improved the visibility of the MC and PL for M3M evaluation. Compared to the original CBCTs, these AI-enhanced low-dose images did not significantly affect risk assessments, treatment strategies, or patient management decisions, and were largely indistinguishable from original images.
**Image quality (quantitative accuracy of voxel values)**								
Yong et al.^27^	2021	To enhance CBCT images for direct quantitative measurement of bone mineral density	Cycle-GAN combined with a multi-channel U-Net	800 pairs of MDCT and CBCT slices from a BMD calibration phantom with and without 3 calcium hydroxyapatite densities	MAD, PSNR, NCC, SSIM, SNU, and linearity	NA	MAD: 203.5, PSNR: 23.9, SSIM: 0.87, NCC: 0.87, linearity: 0.83 The generated CBCT images closely matched CT in BMD distribution, contrast, and anatomical structure.	The model enables direct, accurate, and quantitative measurement of BMD from CBCT images.
Kang et al.^28^	2023	To improve the quality and Hounsfield unit (HU) accuracy of CBCT	Patchwise contrastive learning-based GAN	Unpaired 6000 CT and 6000 CBCT slices from 24 patients and paired 1500 CT and CBCT slices from 6 patients	FID, PSNR, MAE, RMSE, SNU, structure score	NA	Best FID (42.13), PSNR (26.74), MAE (63.04), RMSE (156.02), and structure score (0.88). Significant reduction in HU discrepancies and artifacts compared to baseline models. Highest correlation with ground truth CT images in line profile and HU histograms	The proposed model enhanced CBCT image quality and HU accuracy, while robustly preserving anatomical structures.
**Image quality (artifact reduction)**								
Wang et al.^29^	2019	To remove ring artifacts from CBCT images	GAN with unidirectional relative total variation (URTV) loss	101 000 CT image slices with synthetic ring artifacts and 20 CBCT images with real ring artifacts	PSNR and SSIM	NA	PSNR: 30.31 SSIM: 0.913	The combination of polar coordinate transformation, GAN with perceptual loss, and URTV loss effectively removes ring artifacts from CBCT images.
Sha et al.^30^	2024	To remove ring artifacts from CBCT images	U-shaped Transformer	5882 CT images from 2016 low-dose CT Grand Challenge and 500 CBCT images from MICCAI 2023 Challenge	PSNR, SSIM, block TV and block coefficient of variance	NA	The model outperformed classical approaches in both simulated and real CBCT data, achieving highest PSNR (48.59) and SSIM (0.99) scores. Block TV and block CV metrics in regions of interest were closest to the reference images, showing best local homogeneity and preservation of textures.	The dual-domain Transformer with a custom loss function effectively removed ring artifacts from CBCT images while preserving fine anatomical details.
Choi et al.^31^	2025	To reduce metal artifacts in CBCT images	Latent diffusion model with an improved normalized metal artifact reduction framework	12 993 paired image slices with and without metal artifact from a phantom and synthetically created artifact maps added to metal-free images from patients	RMSE, PSNR, SSIM, and local standard deviation	NA	RMSE improved from 34.78 to 19.30 PSNR improved from 49.3 to 54.3 SSIM improved from 91.2 to 97.2 Standard deviation in soft tissue regions reduced	The proposed algorithm effectively reduces metal artifacts in dental CBCT, enhances image quality, and preserves anatomical details.
Zhang et al.^32^	2025	To reduce CBCT metal artifacts	Coupled diffusion models	263 CBCT scans from 135 patients	FID, KID, NIQE, and PI	Unclear visual assessments (unknown criteria for scoring) by 4 radiologists	*Objective* The proposed models achieved superior results on FID, KID metrics compared to other existing models. *Subjective* Radiologists ranked the proposed models highest for image quality compared to other existing models.	The proposed models significantly improved metal artifact reduction in CBCT images, outperforming existing models.
Wang et al.^33^	2025	To reduce metal artifacts in CBCT images by integrating intraoral optical scanning data with CBCT projection data	A guided-diffusion model	17 metal-free CBCT and 4 CBCT scans with metallic dental materials and corresponding intraoral scans	Mean absolute difference in masked regions	NA	Mean absolute difference Proposed method: 0.0364 GAN-based method: 0.0640 Linear interpolation: 0.0785	The model, guided by intraoral data, provides effective suppression of metal artifacts and improved image quality.
Wang et al.^34^	2026	To reduce metal artifacts in CBCT images	Two-stage diffusion framework	149 CBCT scans (118 without artifacts and 31 with artifacts)	PSNR, SSIM, LPIPS, DICE, and IoU.	Image quality assessment by 4 radiologists	PSNR: 34.79, SSIM: 0.927, LPIPS: 0.058, DICE: 0.758, IoU: 0.675 Best in artifact reduction: 66.67% Best in structure preservation: 68.33%	The proposed 2-stage diffusion model with structure-aware edge extraction and segmentation-guided sampling significantly improves CBCT metal artifact reduction.
Oliveira et al.^35^	2025	To reduce metal artefacts originating from the exomass in CBCT	Conditional denoising diffusion probabilistic model	400 scans with different FOVs and different number and material of dental implants, taken using 4 CBCT machines	CNR	Three oral radiologists scored image quality and hard tissue visualization using a 5-point scale	*Objective* Significantly higher CNR than both ground truth and original images, regardless of implant material, quantity, and CBCT machine. *Subjective* Most scores for corrected images were at 4 or 5 (possibly/definitely superior to original).	The model demonstrated promising performance in correcting exomass-related metal artefacts in CBCT scans.
Candemil et al.^36^	2026	To reduce CBCT artefacts caused by high-density dental materials	Tailored GAN	Images of a partially edentulous human mandible with 40 endodontically treated teeth (gutta-percha, sealer, and cobalt-chromium posts) inserted into sockets and 2 adjacent titanium implants	SSIM, PSNR, MAE, RMSE, noise, and SNR.	Four endodontists rated diagnostic confidence for assessing dental root compromise, artefact interference, and dental/bone preservation using a 5-point scale	*Objective* GAN-corrected images showed significantly higher SSIM and PSNR, and lower MAE and RMSE compared to uncorrected images. Corrected images had lower noise and higher SNR, regardless of artefact severity. *Subjective* Observers rated GAN-corrected images higher for diagnostic confidence and lower for artefact interference.	The GAN-based approach significantly improves CBCT image quality by reducing artefacts from dental materials, enhances diagnostic confidence, and preserves anatomical detail better than conventional MAR or other deep learning models.
Ko et al.^37^	2021	To reduce both rigid and non-rigid motion artifacts in CT and CBCT images	A deep learning network consists of 10 AttBlocks	Fan-beam CT teeth dataset (2-DoF rigid motion), CQ500 dataset (2-DoF rigid motion, real head scans), CBCT teeth dataset (6-DoF rigid motion)	PSNR and SSIM	NA	FBCT teeth (2-DoF) PSNR: 37.35 SSIM: 0.94 CQ500 (2-DoF) PSNR: 29.03 SSIM: 0.91 CBCT teeth (6-DoF) PSNR: 38.21 SSIM: 0.93	The proposed model effectively and efficiently reduced both rigid and non-rigid motion artifacts in CT images from various systems, without needing projection data or external tracking.
Park et al.^38^	2022	To correct shading artifacts in CBCT	Two-stage GAN	12 100 CBCT image slices from 20 patients and 11 422 MDCT image slices from 28 patients	Mean HU values in bone regions and DICE for bone segmentation	NA	Mean Bone HU Original: 1162 ± 472 Corrected: 1220 ± 565 DICE Original: 0.81 Corrected: 0.91	The proposed unpaired-paired learning approach effectively corrected shading artifacts in CBCT, allowing more accurate 3D bone segmentation.
Park et al.^39^	2025	To reduce shading artifacts when transforming CBCT images into high-quality, MDCT-like images	Cycle-GAN-based generative adversarial network	12 100 CBCT image slices from 20 patients and 11 422 MDCT image slices from 28 patients	ARR, ARSR, RMSE, SSIM, and DICE	NA	ARR: 97.58% ARSR: 98.74% RMSE: 0.024 SSIM: 0.978- 0.981 DICE: 0.889-0.965	The fine-tuning method using style transfer and human feedback substantially reduces shading artifacts in CBCT-to-MDCT image transformation.
**Image simulation (simulation without corresponding resources)**								
Kirkwood et al.^40^	2025	To generate realistic synthetic bitewing radiographs of the right molar region	Three diffusion models with a U-Net architecture	200 images labeled by one dentist (Model 1) and 200 images consisting of 57 expert selected images and duplicates (Models 2 and 3)	FID and KID	A dentist graded synthetic radiographs using a 5-point scale (1 = highly realistic, 5 = unrealistic)	*Objective* Model 3 consistently achieved the lowest (best) FID and KID scores, indicating the highest similarity to real radiographs. Model 2 performed better than Model 1 but worse than Model 3. *Subjective* Model 1: <15% realistic Model 2: 22% realistic (expert curation) Model 3: 90% realistic (expert curation and model refinement)	Diffusion models, when refined with expert-informed data curation and architectural optimization, can generate highly realistic synthetic radiographs. Notably, the resolution of synthetic images was lower than original radiographs.
Kim et al.^41^	2021	To generate synthetic high-resolution lateral cephalometric radiographs	Progressive growing generative adversarial network (PGGAN)	15 833 lateral cephalometric images from the Kooalldam Dental Hospital in Korea	SNR	13 dental students, residents, orthodontists distinguished real from synthesized images 2 orthodontists traced 42 anatomical landmarks on synthesized images	No significant difference between real (23.54) and synthesized (26.46) images Both orthodontists and non-orthodontists had difficulty distinguishing real from synthesized images (mean accuracy: 49.2% non-orthodontists, 67.1% orthodontists) Mean difference between orthodontists for 42 points: 2.08 mm. Most landmarks <2 mm difference; largest at occlusal plane (5.95 mm)	PGGAN can generate highly realistic cephalometric X-ray images with quality comparable to real images for most anatomical and diagnostic features.
Huang et al.^42^	2021	To synthesize enhanced 2D cephalometric images from CBCT scans	pix2pixGAN (cephalogram synthesis); ESRGAN and pix2pixGAN (super resolution)	Publicly available CT datasets (CQ500), simulated CBCT projections, and benchmark datasets (ISBI)	PSNR (cephalogram synthesis and super resolution)	NA	Cephalogram synthesis PSNR: 33.8 Super Resolution PSNR: 32.5	Synthetic cephalograms with enhanced contrast and resolution can be reliably generated from CBCT data using the proposed method.
Schoenhof et al.^43^	2024	To generate synthetic panoramic radiographs	GANs	9599 panoramic radiographs	NA	54 dentists, maxillofacial surgeons, residents, trainees, and 33 dental students assessed images as real or synthetic and evaluated the quality of synthetic images using a 10-point scale.	Overall correct rate of identifying synthetic images: 78.2% Overall correct rate of identifying real images: 82.5% Median image quality score for synthetic images: 6/10	GANs can create synthetic panoramic radiographs that are often indistinguishable from real images, even by experienced professionals.
Pedersen et al.^44^	2025	To generate synthetic panoramic radiographs	Deep convolutional generative adversarial network (DCGAN) based on Wasserstein loss with gradient penalty (WGAN-GP)	2322 panoramic radiographs (open dataset MICCAI-DENTEX) cropped to focus on the dentoalveolar region	FID and t-SNE visualization	A dentist rated 100 generated images using a 5-point scale for realism, clarity/sharpness, anatomical features, and artifact absence.	*Objective* FID: 312.8-364.5 t-SNE: generated images clustered closer to real images than noise. *Subjective* Most synthetic images showed moderate anatomical depiction but were impaired by artifacts. Model trained on non-denoised data had highest scores for mandibular canal, trabecular bone while model trained on denoised data had better clarity, sharpness, and overall realism.	WGAN-GP can generate synthetic cropped panoramic radiographs with moderate realism and anatomical detail, but quality is not yet sufficient for clinical use.
Yu et al.^45^	2025	To generate high-fidelity synthetic panoramic radiographs with accurate tooth structures	Diffusion-model-based framework (ToothMaker)	3657 annotated panoramic images from multiple sources	FID, IS, and MS-SSIM	NA	ToothMaker outperformed all baselines in all metrics: FID: 72.91 (ToothMaker) vs. 124.12-275.69 (others) IS: 2.85 (ToothMaker) vs. 2.25-2.71 (others) MS-SSIM: 0.63 (ToothMaker) vs. 0.52-0.56 (others)	ToothMaker enables realistic, high-fidelity synthetic panoramic image generation.
Waqas et al.^46^	2025	To generate high-resolution, anatomically realistic synthetic panoramic radiographs	Four GAN models	4777 images from clinical centers in Pakistan, Thailand, and the United States	Mean average precision	Dentists and radiologists evaluated teeth, maxilla, mandible, sinuses, mandibular canals, and clarity using a 5-point scale	*Objective* The overall mAP score ranges from 0% to 53.7% across the 4 models. *Subjective* Average score Real images: 76.67% Models: 25.62%-43.24%	The GAN-based pipeline enables ethical creation of synthetic images, suitable for augmenting datasets.
Fukuda et al.^47^	2024a	To generate high-quality panoramic radiographs including dentigerous cysts	StyleGAN3	459 panoramic radiographs with cystic lesions (suspected dentigerous cysts), from 430 patients	FID, KID, PR, and IS	Distinguishing between real or generated images and rating 4 findings (cyst boundary, unilocular nature, root resorption, mandibular canal visibility) using a 4-point scale by 3 oral radiologists	*Objective* FID: 199.28; KID: 0.14; Precision: 0.0047; Recall: 0; IS: 2.48 *Subjective* Radiologists could not distinguish 82.3% real from generated images. Generated images were significantly inferior to real images for cyst border, unilocular nature, and mandibular canal visibility, but no significant difference for root resorption.	StyleGAN3 generated high-quality panoramic radiographs of dentigerous cysts, with some images indistinguishable from real ones by specialists.
**Image simulation (image-to-image translation)**								
Ryu et al.^48^	2024	To generate high-quality synthetic CT (sCT) images from CBCT for accurate segmentation of the entire upper airway	CycleGAN	33 cases with both CT and CBCT scans acquired at different time or in different position, from Severance Hospital, Yonsei University	MAE, PSNR, SSIM, and DICE	NA	MAE decreased from 161.60 to 100.54 PSNR increased from 22.33 to 28.65 SSIM increased from 0.617 to 0.865 DICE for airway segmentation improved from 0.849 (CBCT) to 0.960 (sCT), nearly matching CT (0.966)	The proposed model effectively denoised CBCT and generated CT-like images, enabling precise and automated segmentation of the entire upper airway, including the complex nasal cavity.
Fukuda et al.^49^	2024b	To enhance the visibility of soft tissues on CBCT images	CycleGAN with a 9-block ResNet generator and 70x70 PatchGAN discriminator	Training: 60 904 CT images (bone and soft-tissue windows) from 200 patients Internal test set: 4272 CBCT images from 10 patients (for normal anatomical structures) External test set: 1485 CBCT images from 10 patients (for cystic lesions)	Histogram analysis of pixel values for anatomical structures and cystic lesions	3 oral radiologists evaluated the visibility of the lateral pterygoid muscles, medial pterygoid muscles, submandibular glands, parotid glands, and submandibular and submental lymph nodes using a 4-point scale	*Objective* Anatomical structures Synthetic CBCT images showed significant shifts in voxel intensity toward CT-like values. Mean pixel values for soft tissues in synthetic CBCT closely matched CT, and were higher than original CBCT. Cystic lesions Synthetic CBCT showed limited improvement; pixel value distribution did not match CT as closely. *Subjective* Radiologists’ scores for visibility were significantly higher for synthetic CBCT than original CBCT, especially in the submandibular region and floor of the mouth, but not equal to CT.	Image synthesis using CycleGAN significantly improved the visibility of soft tissue on CBCT, especially from the submandibular region to the floor of the mouth while improvement for cystic lesions was limited.
Fukuda et al.^50^	2024c	To convert non-contrast CT (non-CECT) images into synthetic contrast-enhanced CT (CECT) images in the internal jugular region	CycleGAN	25 pairs of CECT and non-CECT scans from patients with head and neck cancers from Aichi Gakuin University Dental Hospital	PSNR and SSIM	Three oral radiologists distinguished synthetic images from original images and distinguished lymph nodes from blood vessels on synthetic CECT and original CECT and non-CECT images	*Objective* PSNR was higher for non-CECT images, SSIM was higher for CECT images. *Subjective* Radiologists could not distinguish synthetic CECT images from original images (accuracy from 52.5% to 58.8%); both synthetic and original CECT images allowed almost perfect discrimination (AUC: 1.0) between lymph nodes and blood vessels while non-CECT images had lower performance (AUC: 0.72).	The cycleGAN model can generate synthetic CECT images from non-CECT images with sufficient quality for distinguishing lymph nodes from blood vessels.
Ha et al.^51^	2025	To generate T2-weighted (T2w) MRI from proton density-weighted (PDw) images in TMD patients	TransUNet-SegGAN	7226 MRI slices from 178 TMD patients, including bilateral PDw and T2w sagittal images in both open- and closed-mouth positions, from Yonsei University Dental Hospital	SSIM, LPIPS, and FID Segmentation performance: Dice score, precision, recall, and Hausdorff distance	Mean opinion score by 3 oral radiologists	*Objective* SSIM 82.28%, LPIPS 2.46, FID 23.85 (best among compared models) Disc Segmentation: Dice score 78.93%, precision 93.9%, recall 89.97%, Hausdorff distance 0.80 mm *Subjective* Score: 4.58 (highest) superior in all critical diagnostic aspects (disc position/structure, effusion, bone marrow signal, overall quality)	The model can generate high-quality T2w MR images from PDw images, especially in the TMJ disc region, and provides robust automatic disc segmentation.
**Image simulation (post-treatment prediction simulation)**								
Park et al.^52^	2022	To generate simulated 3D post-orthodontic facial changes using CBCT	Conditional GAN with U-net generator and PatchGAN discriminator	312 pairs of pre-treatment (T1) and post-treatment (T2) CBCT scans from adults who had orthodontic treatment	Mean absolute distances between predicted and real T2 surfaces in 6 perioral/anatomical landmark regions	56 orthodontists distinguished real from predicted T2 face and rate realism	*Objective* Mean error: 1.2 ± 1.0 mm (across all landmarks). 80.8% of predictions had error ≤2 mm. *Subjective* More than 50% of orthodontists could not distinguish real from predicted images. After revealing image types, 67% selected real T2; 32% selected predicted T2.	The model can reliably predict individual 3D post-orthodontic facial outcomes with clinically acceptable accuracy and many orthodontists could not distinguish them from real post-treatment faces.
Liang et al.^53^	2020	To generate reconstructed mandible in patients with mandibular defects on CBCT images for surgical planning	DCGAN	14 278 CBCT image slices of normal mandibles from 50 patients without mandibular defects and 6 scans from patients with defects	NA	Visual and quantitative assessment of anatomical plausibility and smoothness of transition between reconstructed and natural regions	The system produced highly realistic and anatomically plausible reconstructions. There was a smooth transition between reconstructed and existing natural bone.	The model enabled realistic and individualized 3D reconstructions of the mandible.
**3D reconstruction**								
Mao et al.^54^	2024	To reconstruct CBCT images from highly undersampled coded aperture projections	GAN-U-Net	102 CBCT scans using coded apertures with transmittance levels of 5%, 10%, and 15% (95%-85% of the x-ray projections were blocked)	PSNR and SSIM	NA	PSNR: 36.4, 42.5, and 39.0 at 15%, 10%, and 5% sampling rates SSIM: 0.98 at all sampling rates	The model enables CBCT reconstruction from highly undersampled coded aperture data, reducing radiation dose (up to 95% fewer projections) without compromising image quality.
Minhas et al.^55^	2024	To reconstruct 3D images of maxillary impacted canines from 2D panoramic radiographs	Pan2CBCT based on X2CT-GAN framework	123 pairs of panoramic and pseudo-3D images generated from CBCT scans of 74 patients with and 49 without impacted canines from Peking University Hospital	SSIM and Accuracy of predicting impacted canine position in buccal/middle/lingual and mesial/distal directions	NA	Mean SSIM: 0.71 (range 0.63-0.84) Accuracy of canine position Buccal: 64% Middle: 33% Lingual: 12% Mesial: 62% Distal: 11%	While the network generated pseudo-3D images from 2D panoramic X-rays with moderate similarity, its accuracy for locating impacted canines is insufficient for reliable treatment planning.
Parida et al.^56^	2025	To reconstruct a high-fidelity, continuous 3D volumetric model of the oral cavity from a single 2D panoramic radiograph	ViT-NeBLa	623 CBCT scans with synthetic panoramic images generated via physically accurate simulation	PSNR, SSIM, and LPIPS	NA	*Objective* PSNR: 23.48 ± 0.78 SSIM: 74.93 ± 2.56 LPIPS: 0.4204 ± 0.0093	ViT-NeBLa enables high-quality, patient-specific 3D dental volume reconstruction from a single panoramic X-ray.
**Automated interpretation and reporting**								
Gao et al.^57^	2025	To diagnose osteonecrosis of the jaw (ONJ) from panoramic radiographs, segment lesion areas, recommend treatment plans, provide automated radiology reports, and support clinicians through interactive Q&A	Swin Transformer for ONJ detection and classification; U-Net for lesion segmentation; Qwen2-VL large language model fine-tuned for ONJ-specific natural language Q&A	366 ONJ-positive patients with panoramic radiographs and medical records and unclear number of ONJ-negative patients	AUC, accuracy, precision, recall, F1, Dice, IoU, BLEU, ROUGE, METEOR	2 senior and 2 junior OMFSs vs. ONJ-TS system, scored by 3 OMFS experts	*Objective* AUC: 0.975, Accuracy: 0.924, Precision: 912, Recall: 0.960, F1-score: 0.935, DICE: 0.930, IoU: 0.868, BLEU-1 to 4: 0.526-0.672, METEOR: 0.844, ROUGE-1 to L = 0.665-0.735 *Subjective* Accuracy score ONJ-TS: 0.960 Junior OMFSs: 0.884 Senior OMFSs: 0.996	ONJ-TS accuracy exceeded junior OMFSs and was comparable to senior OMFSs.
Dasanayaka et al.^58^	2025	To generate detailed radiology reports, detect major dental diseases, and interact with patients via a Q&A system	Blip-2-based image caption generator and Llama 3 8B for radiology report generation	Image and caption dataset, radiology reports dataset, Sinhala language corpus, and Sinhala dental Q&A dataset	Diagnostic accuracy, ROUGE score, custom DDSLU benchmark for Sinhala Q&A	Subjective scoring by 1 oral radiologist, 1 senior dentist, and 1junior dentist using a 10-point scale	*Objective* Overall diagnostic accuracy: 81.3% Dental caries: 87.9% Impacted teeth: 89.7% Bone loss: 88% Periapical lesions: 81.8% ROUGE-1 score: 72.7% *Subjective* Weighted average subjective rating: 7.5/10 for report quality Sinhala Q&A weighted average score: 6.1/10	The proposed AI system demonstrates automation of dental panoramic radiology report generation and dental Q&A, including in the Sinhala language.

Abbreviations: ASSD = average symmetric surface distance; CBCT = cone-beam CT; CNN = convolutional neural network; DICE = dice similarity coefficient; ESPCNN = efficient sub-pixel convolutional neural network; FID = Fréchet inception distance; GAN = generative adversarial network; GP = general practitioner; HAT = hybrid attention transformer; HU = Hounsfield unit; IoU = intersection over union; IS = inception score; KID = kernel inception distance; LDM = latent diffusion model; LPIPS = learned perceptual image patch similarity; LTE = local texture estimator; MAD = mean absolute difference; MAE = mean absolute error; MLP = multilayer perceptron; MSE = mean squared error; NCC = normalized cross correlation; OMFS = oral and maxillofacial surgeon; PSNR = peak signal-to-noise ratio; RMSE = root mean squared error; SNR = signal-to-noise ratio; SNU = spatial nonuniformity; SRCNN = super-resolution convolutional neural network; SRGAN = super-resolution generative adversarial network; SSIM = structural similarity index measure; TMD = temporomandibular disorder; ViT = vision transformer.

The most common applications of DMFR-specific Gen-AI are image quality enhancement (*n* = 24). Gen-AI models have been developed to improve the spatial resolution of periapical, panoramic, and CBCT images; denoise low-dose CBCT; reduce blur and correct distortions in panoramic radiographs; enhance CBCT image quality; standardize style across panoramic radiographs from different devices; improve the accuracy of Hounsfield unit (HU) measurements in CBCT; quantify bone mineral density from CBCT; and remove various artifacts, including those caused by metal, patient motion, ring, and shading, in CT and CBCT scans (Table 4).

The second most common application of DMFR-specific Gen-AI is image simulation (*n* = 14), which includes generating radiographs without corresponding resources (eg, bitewing, panoramic, and cephalometric images), performing image-to-image translation (eg, creating synthetic CT from CBCT, high-quality micro-CT from CBCT, contrast-enhanced CT from non-contrast CT, and T2-weighted MRI from proton density-weighted MRI for temporomandibular joint disc segmentation), and simulating post-treatment predictions (eg, reconstructing 3D mandibular morphology from CBCT and predicting 3D post-orthodontic facial changes from CBCT) (Table 4).

Individual studies (*n* = 5) have proposed Gen-AI models for a range of applications including reconstructing 3D CBCT from incomplete projections, reconstructing 3D images of impacted canines from 2D panoramic images, and creating 3D volumetric models of the oral cavity from 2D panoramic images. Additionally, Gen-AI models have been developed for the automated interpretation and reporting of osteonecrosis of the jaw (ONJ) and various dental conditions from panoramic radiographs (Table 4).

Nearly all included studies (95.3%) reported objective quantitative evaluation metrics, such as peak signal-to-noise ratio (PSNR), SSIM, MSE, Fréchet inception distance (FID), learned perceptual image patch similarity (LPIPS), mean absolute error (MAE), RMSE, dice similarity coefficient (DICE), and others, to assess the performance of their Gen-AI models for various DMFR tasks. About half of the studies (58.1%) incorporated subjective clinician assessments (eg, by dentists or oral radiologists) using scoring systems or visual grading to judge clinical image quality, realism, diagnostic confidence, or usefulness. Twenty-three studies (53.5%), especially those focused on image enhancement and synthetic image generation studies, included both objective and subjective analysis (Table 4).

The risk of bias in the included studies was evaluated on 4 major domains of bias (dataset details, reporting of Gen-AI model architecture and training strategies, reliability of performance evaluation methods, and accessibility of code and developed models). In the dataset domain, 7 studies (16.3%) were rated as having a “moderate” risk of bias due to insufficient details regarding the source or number of images used. For the Gen-AI model architecture domain, all studies were rated as “low” risk of bias, as they provided clear descriptions of the model architecture and training strategies adopted. In the performance evaluation domain, 20 studies (46.5%) were rated as “moderate” risk of bias because they assessed performance using only objective or subjective methods rather than both. Regarding the accessibility, 35 studies (81.4%) were rated as “high” risk of bias, as they did not provide valid access to the code or models for their proposed Gen-AI systems. A summary of the risk of bias assessment for all included studies is provided in Table S3.

## Discussion

This study aimed to systematically review the current development and application of DMFR-specific Gen-AI networks. Since 2019, a growing number of studies have proposed DMFR-specific Gen-AI networks for a wide range of DMFR tasks. Of the 43 included studies, the majority focused on improving image quality, particularly through artifact reduction and spatial resolution enhancement in CBCT. In addition, Gen-AI models have also demonstrated promising capabilities in generating highly realistic synthetic radiographs, simulating post-treatment anatomical outcomes, reconstructing 3D structures from limited data, and automating interpretation and reporting. While nearly all studies reported objective quantitative performance metrics, only about half incorporated clinician-based subjective assessments, highlighting a gap in clinical validation. Notably, most studies exhibited a high risk of bias regarding accessibility as code and trained models were rarely made available for independent validation. These findings highlight the significant potential of Gen-AI to advance DMFR imaging, while emphasizing the need for more robust clinical validation, transparency, and open access to facilitate safe and reliable clinical application.

### Image quality (spatial-resolution)

Several studies have developed and compared multiple deep learning-based Gen-AI models to enhance the resolution and quality of dental radiographs using both private and open-source datasets that include pairs of original images and corresponding images that have been intentionally reduced in quality (ie, artificially degraded images). Moran et al.^16^ developed super-resolution generative adversarial network (GAN) models using transfer learning strategies with medical and non-medical image datasets to enhance the spatial resolution of periapical radiographs. The model trained with transfer learning generated high-resolution periapical images with more defined edge details and fewer blur effects. Mohammad-Rahimi et al.^17^ reported that the model trained with a local texture estimator approach achieved the highest performance in reconstructing enhanced image details. Çelik et al.^18^ trained a deep learning-based Gen-AI model using pairs of original high-resolution panoramic radiographs and sets of the same images that had been intentionally downscaled to lower resolutions with fewer pixels. Although their model achieved the best overall performance among those tested, the performance of all models decreased as the image resolution was reduced. This finding suggests that Gen-AI models’ ability to enhance image quality may be limited when working with images that have very low resolution.

Recent studies have also investigated the potential of Gen-AI models to enhance CBCT images using paired CBCT and micro-CT datasets. Chen et al.^19^ developed a variation-driven neural network model that employed a variation-based distance metric and an end-to-end training strategy to generate high-resolution micro-CT-like images from CBCT scans, allowing improved visualization of tooth structures, particularly root canal systems. In another study, deep learning-based super-resolution models were trained with paired CBCT and micro-CT images using an edge loss strategy to improve the spatial resolution and structural accuracy of CBCT scans.^20^ The trained models demonstrated high performance in objective image quality metrics, visual assessments, and 3D root canal reconstruction, generating image quality that closely approaches that of micro-CT. Such models can significantly enhance the diagnostic value of CBCT for endodontic indications by enabling clearer visualization of fine root canal structures.

Sabry et al.^21^ introduced a self-adaptive multi-objective metaheuristic algorithm combined with a specialized pathology-aware latent diffusion model (DentoSMART-LDM) to not only improved the quality of panoramic radiographs but also generated synthetic radiographic images displaying 12 conditions (eg, dental caries, periodontal bone loss, apical periodontitis, root resorption, impacted teeth, cystic lesions) to support automated detection and classification systems. DentoSMART-LDM showed stable performance across 7 independent test datasets, highlighting its potential for screening a range of dental and jawbone conditions, particularly in underserved communities.

### Image quality (improvement of image geometry and projection)

Some studies have proposed Gen-AI models to improve image geometry and projection in dental radiography. Kim et al.^22^ demonstrated that a trained Pix2Pix network could significantly recover and sharpen blurred structures in panoramic radiographs, with clinicians reporting the greatest improvements in images affected by blur, especially in the anterior teeth region, although the model was less effective for images with noise or combined blur and noise. Kolukisa et al.^23^ found that Pix2Pix consistently outperformed CycleGAN and RegGAN in correcting a range of common distortions in panoramic radiographs, regardless of distortion type. These findings showed the potential of GAN-based models, particularly Pix2Pix, to enhance the visual quality of panoramic radiographs.

In another study, Kim et al.^24^ developed a CycleGAN model trained on unpaired panoramic radiographs from 2 different equipment types to harmonize image styles and achieve standardization across devices. This harmonization approach could address a key challenge of developing AI systems that perform reliably with non-uniform radiographic images. By standardizing pixel or voxel intensity distributions across equipment, such image harmonization can serve as an effective pre-processing method to improve the reliability of high-dimensional feature selection and enhancing the overall stability of model performance, particularly crucial for radiomic analysis.^59^^,^^60^

### Image quality (noise reduction)

Ensuring patient safety and minimizing unnecessary radiation dose exposure are fundamental goals in radiographic imaging.^61^ Strict adherence to radiological protection principles, including ALARA (As Low As Reasonably Achievable), ALADA (As Low As Diagnostically Acceptable), and ALADAIP (As Low As Diagnostically Acceptable being Indication-oriented and Patient-specific), is crucial to minimize patient radiation exposure while ensuring diagnostic accuracy.^62^ Some studies have aimed to improve low-dose CBCT images by reducing noise, making their quality similar to that of high-dose scans. Hegazy et al.^25^ developed a U-Wasserstein GAN model using a transfer learning strategy, first pre-training on public-domain medical CT image pairs and then fine-tuning with limited high-dose and low-dose CBCT image pairs, to denoise low-dose CBCT images. The trained model outperformed conventional CNN models by avoiding over-smoothing effects and efficiently suppressing noise in low-dose CBCT images. Low-dose CBCT may reduce the visibility of certain anatomical structures relevant to impacted third molar evaluation.^63–65^ To address this, Zhang et al.^26^ developed a CycleGAN-based model using paired standard- and low-dose CBCT images to enhance the image quality of low-dose CBCT scans for evaluating impacted mandibular third molars. The results demonstrated that enhanced low-dose CBCT images significantly improved the visibility of the mandibular canal and periodontal ligament space, achieving clarity and structural fidelity comparable to standard-dose images. Importantly, the enhanced images did not significantly affect clinicians’ risk assessments, treatment strategies, or referral decisions, regardless of their qualifications.

### Image quality (quantitative accuracy of voxel values)

Yong et al.^27^ developed a hybrid deep-learning model combining Cycle-GAN and U-Net using paired training data of quantitative CT and CBCT images to directly and quantitatively measure bone mineral density from CBCT images. The results showed that model demonstrated improved contrast, uniformity, and anatomical accuracy of bone images under various scanning conditions. The model can substantially enhance the quantitative accuracy of bone mineral density measurements from CBCT images. Kang et al.^28^ developed a patchwise contrastive learning-based GAN model trained on paired CBCT and CT datasets to generate CT-like images from CBCT scans, thus enhancing CBCT image quality and aligning its gray values with HU measurements. By enabling more accurate HU measurements, the model ensures that the gray level differences in the generated CT-like images more faithfully reflect the true attenuation differences of the exposed object, leading to enhanced contrast resolution and addressing a key limitation of CBCT, which does not natively provide standardized HU values. This approach may allow for more accurate HU quantification from enhanced CBCT images.

### Image quality (artifact reduction)

Several studies proposed Gen-AI models using datasets of paired synthetic artifact-corrupted and original clean CBCT images to remove various types of artifacts in CBCT images. Wang et al.^29^ developed a GAN model to effectively remove artifacts while preserving critical texture information. Sha and Li^30^ introduced a Transformer-based model using a dual-domain information fusion strategy to remove ring artifacts from CBCT images. Their models achieved superior image quality and evaluation metrics compared to classical methods, effectively eliminating ring artifacts while maintaining image details.

Choi et al.^31^ developed a metal artifact reduction algorithm for CBCT using a conditional latent diffusion model within a modified normalized framework, significantly outperforming classical and CNN-based methods. Zhang et al.^32^ introduced a coupled diffusion model that trained on both artifact-degraded and clean CBCT images, which outperformed conventional methods in both objective metrics and visual quality, especially in severe cases. Wang et al.^33^ proposed a guided-diffusion model integrating CBCT projection data with intraoral optical scans to correct metal artifacts, suppressing artifacts and better preserving tooth shape and structure compared to GAN-based and linear interpolation methods. Wang et al.^34^ further developed a structure-preserving 2-stage diffusion model using tooth contour supervision from intraoral scan-CBCT fusion to extract clean edge maps and guide artifact reduction, achieving superior artifact reduction and dental structure preservation compared to traditional interpolation-based, supervised, and unsupervised deep learning approaches.

Some studies have focused specifically on artifacts caused by dental implants and other high-density dental materials in CBCT scans. Oliveira et al.^35^ developed a deep learning model to reduce metal artifacts in CBCT scans, specifically focusing on exomass-related artifacts caused by dental implants. Using paired CBCT images with and without dental implants, their model significantly improved hard tissue visualization, overall image quality, and contrast-to-noise ratio compared to both original artifact-affected images and ground truth images, demonstrating effective correction of exomass-related metal artifacts. Candemil et al.^36^ introduced a GAN-based model trained on paired CBCT images with and without high-density dental materials to address artifact reduction. Their approach resulted in corrected images with significantly higher diagnostic confidence, reduced artifact interference, and favorable preservation of anatomical structures compared to the original images, minimizing artifacts from various high-density dental materials in CBCT scans.

Some studies have specifically focused on motion and shading artifacts in CBCT imaging. Ko et al.^37^ developed an attention-based deep residual network trained on motion-corrupted and ground-truth CT and CBCT image pairs across various simulated motion scenarios to reduce both rigid and non-rigid motion artifacts. Their model outperformed both Wasserstein GAN-based and standard deep residual network methods, compensating for diverse motion artifacts in CT and CBCT images. Park et al.^38^ introduced a 2-stage GAN model with an unpaired-paired learning strategy to reduce shading artifacts in CBCT images. This approach significantly reduced shading and bone-like artifacts, preserved morphological structures, and significantly improved bone segmentation accuracy, therefore enhancing image quality and enabling more precise 3D bone segmentation. Park et al.^39^ further developed a fine-tuned Cycle-GAN model to reduce shading artifacts, preserved local morphological structures, and improved overall image quality compared to conventional Cycle-GAN and non-selective training approaches, allowing the generation of high-resolution, artifact-free images.

### Image simulation (simulation without corresponding resources)

Recent studies have demonstrated the potential of Gen-AI to create highly realistic synthetic dental radiographic images. Kirkwood et al.^40^ developed a diffusion-based U-Net model to generate synthetic bitewing radiographs of the right molar region, although the resolution of these images was lower than that of the original radiographs. Kim et al.^41^ trained a progressive growing GAN using over 15 000 lateral cephalometric radiographs to generate realistic high-resolution cephalometric images. It was reported that both orthodontists (67.1% accuracy) and non-orthodontists (49.2% accuracy) had difficulty distinguishing real from synthetic images. Huang et al.^42^ employed a pix2pix GAN to synthesize high-contrast 2D cephalograms from low-dose CBCT data, achieving high signal-to-noise ratios as well as accurate and efficient automated landmark detection. This model could allow for the generation of cephalometric images directly from CBCT scans, which eliminates the need for additional conventional cephalometric radiographs and reducing patient radiation exposure when a CBCT scan is available.

Some studies have specifically explored the generation of synthetic panoramic radiographs using various Gen-AI architectures and training strategies. Schoenhof et al.^43^ developed a StyleGAN2-ADA model trained on real panoramic radiographs to generate synthetic, non-person-related panoramic images, which experienced professionals and students could not reliably distinguish from authentic images (overall sensitivity 78.2%, specificity 82.5%). Pedersen et al.^44^ implemented a DCGAN with Wasserstein loss and gradient penalty to generate synthetic panoramic radiographs focused on the dentoalveolar region. Yu et al.^45^ introduced the ToothMaker diffusion-based model, which enabled precise control over tooth structure and radiological style, outperforming conventional methods. Waqas et al.^46^ used a high-resolution GAN model with progressive training on multi-source panoramic radiograph datasets, generating synthetic images with fine anatomical detail. Fukuda et al.^47^ developed a StyleGAN3-based model to generate high-quality panoramic radiographs of dentigerous cysts. Oral radiologists were unable to distinguish between real and generated images in 82.3% of cases. Notably, the generated images were significantly inferior to real images for cyst border, unilocular appearance, and mandibular canal visibility.

### Image simulation (image-to-image translation)

Current AI applications in medical imaging are increasingly focused on translating one imaging modality to another, such as generating synthetic CT from CBCT or enhancing MRI contrasts.^66^ These image-to-image translation models, commonly based on advanced GANs and transformers, allow clinicians to obtain missing or higher-quality imaging data without the need for additional scans. Ryu et al.^48^ developed a CycleGAN model using unpaired CT and CBCT image data to generate synthetic CT images from CBCT scans, resulting in enhanced CBCT image quality and improved automatic airway segmentation accuracy. Fukuda et al.^49^ introduced a CycleGAN-based model trained on unpaired CT and CBCT images to improve soft tissue visibility in CBCT scans. The synthesized CBCT images demonstrated significantly improved soft tissue visualization, with voxel intensity distributions and oral radiologist scoring closely approaching those of CT, particularly in the submandibular region and floor of the mouth, although improvement for cystic lesions was limited. In another study, Fukuda et al.^50^ used a CycleGAN model to generate contrast-enhanced CT images from non-contrast CT images of the internal jugular lymph node bearing area. The synthetic contrast-enhanced CT images were visually indistinguishable from original images, and both synthetic and original images achieved perfect differentiation of lymph nodes from blood vessels (AUC = 1), substantially outperforming non-contrast CT images (AUC = 0.72). 3D imaging modalities, including CT, CBCT, and MRI, are commonly used for assessing oral-head and neck neoplasia as well as pathologies of the paranasal sinuses.^67–72^ In dental medicine, MRI also plays an important role in the temporomandibular joint (TMJ) evaluation, especially pathologies of the disc. Ha et al.^51^ developed a transformer-based GAN model using paired proton density-weighted and T2-weighted MRI to generate high-quality T2-weighted images and improve TMJ disc segmentation, achieving superior quantitative results and the highest qualitative scores from oral radiologists (mean score 4.58 out of 5). These cross-modality synthesis techniques have the potential to significantly enhance both the quality and accessibility of various types of diagnostic imaging.

### Image simulation (post-treatment prediction simulation)

Gen-AI networks have been developed to simulate post-treatment anatomical outcomes and reconstruct complex oral-maxillofacial structures. Park et al.^52^ proposed a deep learning-based 3D facial prediction model for post-orthodontic outcomes using a conditional GAN trained on paired pre- and post-orthodontic treatment CBCT datasets, incorporating patient-specific factors such as age, gender, lip incompetency, and incisor movements. The model’s predicted facial images closely matched actual outcomes, with a mean error of 1.2 ± 1.0 mm and 80.8% of errors within the clinically acceptable threshold of 2 mm. Notably, more than half of experienced orthodontists could not distinguish between real and predicted images. Liang et al.^53^ developed a deep convolutional GAN to reconstruct mandibular morphology from 3D CBCT images in patients with mandibular defects caused by tumors or mandibulectomy. The model generated 3D mandibles with natural anatomical features and smooth transitions to unaffected areas, even in challenging cases involving large or midline-crossing defects. Most reconstructions were rated by specialists as indistinguishable from real anatomical structures, demonstrating its potential use for reconstructive surgical planning.

### 3D reconstruction

Recent studies have explored the potential of Gen-AI networks for efficient, lower-radiation 3D reconstructions from limited or routine radiographic imaging. Mao et al.^54^ developed a GAN-based model to predict complete and uniform CBCT projections from incomplete, non-uniform coded aperture data. This model enabled high-quality 3D CBCT reconstruction even when 95% of projections were blocked, therefore significantly reducing radiation dose, accelerating reconstruction time, and improving image fidelity compared to traditional methods. Minhas et al.^55^ introduced Pan2CBCT, a deep generative model trained on paired 2D panoramic radiographs and pseudo-3D CBCT images to assess maxillary impacted canines. While the model achieved favorable accuracy in anatomical position prediction, the image quality was limited by blurring, which restricts its clinical applicability. More recently, Parida et al.^56^ proposed ViT-NeBLa, a hybrid Vision Transformer-CNN model with physics-guided training and learnable hash positional encoding, which could reconstruct 3D dentoalveolar anatomy directly from a single panoramic radiograph. ViT-NeBLa outperformed existing PX-to-3D approaches in image quality metrics, produced sharper anatomical details, and reduced computational cost by 52%.

### Automated interpretation and reporting

Current development in multimodal AI systems have enabled automated, accurate, and accessible dental diagnostics and decision support by integrating imaging and clinical data. Gao et al.^57^ developed the ONJ treatment supporting system, which combines panoramic radiographs and clinical records to assist in diagnosis and treatment planning for ONJ. The system automates diagnosis and lesion segmentation, provides comprehensive treatment planning through interactive Q&A, generates structured radiology reports, simulates treatment outcomes, and offers educational guidance for junior clinicians. Notably, the system achieved diagnostic and treatment accuracy comparable to senior oral and maxillofacial surgeons, which significantly outperformed junior clinicians. Similarly, Dasanayaka et al.^58^ introduced a multimodal AI platform using a Blip-2-based image caption generator trained on panoramic radiographs and a fine-tuned Llama 3 8B LL, further adapted with domain-specific radiology and Sinhala language datasets. This system could automate radiology report generation and enable dental question-answering in both English and Sinhala. It achieved an overall diagnostic accuracy of 81.3%, with high detection rates for dental caries, impacted teeth, bone loss, and periapical lesions, and was rated 7.5 out of 10 by dental professionals. These 2 studies suggest that multimodal AI systems have the potential to support more accurate diagnostics, efficient clinical workflows, and broader access to dental care, though further validation is needed.

### Prospects and challenges of DMFR-specific Gen-AI in clinical practice, education, and research

Gen-AI is rapidly advancing the DMFR field through a range of innovative applications. One of the most promising clinical applications of DMFR-specific Gen-AI models is improving image quality by enhancing spatial resolution, reducing noise, blur, and artifacts, and increasing the visibility of anatomical structures. Advanced GAN and diffusion-based models have been shown to suppress metal and ring artifacts while preserving anatomical structures in CBCT. Additionally, recent studies have shown that GANs, diffusion models, and transfer learning strategies can improve the diagnostic value of periapical, panoramic, and CBCT images, even when acquired using low dose settings.^16–18^^,^^26^ The improved image quality may not only facilitate more accurate diagnosis and treatment planning but also help clinicians to adhere to the ALARA, ALADA, and ALADAIP principles by reducing the need for repeat imaging or higher radiation exposure.

Another promising clinical application of Gen-AI is its capacity for cross-modality image synthesis. Models such as CycleGAN, transformer-based GANs, and variation-driven neural networks have demonstrated the potential to translate CBCT to synthetic CT, enhance MRI contrast, and even generate synthetic contrast-enhanced CT or micro-CT images from routine scans.^19^^,^^48^^,^^50^^,^^51^ This capability allows clinicians to obtain missing or higher-quality imaging information without the need for additional radiation exposure or multiple scan protocols.

In addition, Gen-AI is playing a critical role in data augmentation and the generation of synthetic datasets. Diffusion models, GANs, and advanced control frameworks have shown the ability to generate realistic dental radiographs, including bitewing, panoramic, and cephalometric radiographs, as well as task-specific panoramic radiographs for diagnostic purposes (eg, jaw cysts). For education, these synthetic datasets can provide dental students and specialty trainees increased exposure to a wider range of normal and pathological cases, including rare conditions that are not typically encountered during clinical training. For research, synthetic images can help overcome data scarcity and class imbalance, particularly for rare diseases, and facilitate the development of robust AI algorithms by enabling more comprehensive training and validation on underrepresented conditions. The integration of Gen-AI with multimodal and multitask systems could further enhance diagnostic support by combining imaging data with clinical records to detect major dental diseases, segment lesions, recommend treatment plans, generate automated radiology reports, and provide both clinicians and patients with interactive Q&A support.

Despite these innovative applications, the translation of Gen-AI into routine clinical practice in DMFR faces several challenges. Data quality and diversity remain critical limitations. Due to the difficulty in acquiring paired scans from the same individuals, many Gen-AI models for image enhancement or artifact reduction have been developed using small private datasets containing paired original and artificially degraded images. However, these artificially degraded images may not accurately capture the complexity or variability of real artifacts, which may limit the models’ clinical applicability. Compared to studies using private datasets, those using open-source datasets could access larger volumes of training data. Nevertheless, reusing these open datasets would introduce several major concerns, including ethical, legal, and quality issues, such as missing ethical approvals, unclear licensing terms, uncertain annotation quality, risk of data leakage (eg, duplicate images across training and testing sets), and potential legal liabilities.^73^

Additionally, many Gen-AI models developed for 3D imaging modalities are trained using 2D image slices rather than full 3D volumes in order to increase the amount of available training data. This approach may not fully capture the spatial relationships present in volumetric data, which might reduce the accuracy and robustness of the resulting models in 3D applications. Furthermore, according to the assessed risk of bias in the included studies, nearly half were rated as having a “moderate” risk of bias because they evaluated model performance using only objective metrics without subjective clinical assessment. This may not adequately reflect the true clinical utility or the potential for diagnostic errors in practice. Notably, more than 80% of the included studies did not provide valid access to the code or trained models for their proposed Gen-AI systems, making it difficult to achieve independent validation, ensure reproducibility, and assess the reliability of these models.^74^

Notably, nearly half of the included studies used only objective image quality metrics, without incorporating clinician-based subjective assessment. However, high scores on objective metrics such as PSNR and SSIM do not necessarily indicate that the generated images are of sufficient quality for clinical use. Therefore, clinician-based subjective evaluation remains important to ensure that improvements in image quality metrics translate into actual clinical applicability.

In some studies, while quantitative metrics and subjective clinician evaluations indicate promising results, there remains a risk of subtle artifacts, over-smoothing, or the generation of synthetic features that could mislead clinicians. A controversial issue that may arise is whether AI-enhanced or synthetic images might “beautify” or otherwise alter images in a way that introduces diagnostic errors, especially in critical cases or rare conditions. Ensuring that no unrealistic or hallucinated errors are present in AI-generated images or reports is a crucial prerequisite before these models can be considered safe and reliable for clinical use.

Ethical and legal concerns are also emerging with the use of synthetic data and AI-generated images, including questions of data ownership, patient consent, and regulatory compliance.^75^ Furthermore, the “black box” nature of many deep learning models can make it difficult for clinicians to interpret or trust the outputs, highlighting the need for greater transparency and explainability in Gen-AI systems.

This systematic review has several limitations. First, there is currently no widely accepted or validated quality assessment tool specifically designed for evaluating studies on radiology-specific Gen-AI. In this review, we used customized risk-of-bias assessment criteria adapted from the updated CLAIM checklist, focusing on clarity regarding dataset sources, reporting of Gen-AI model architecture and training strategies, reliability of performance evaluation methods, and accessibility of code and developed models. While these domains were chosen to address key reliability concerns, the effectiveness of the customized criteria in identifying potential risk of bias in DMFR-specific Gen-AI studies remains uncertain. In addition, quantitative synthesis or meta-analysis was not performed due to considerable heterogeneity in the objectives, methodologies, and outcome metrics among the included studies. Despite these limitations, this systematic review provides a comprehensive overview of the current development and performance of DMFR-specific Gen-AI networks, emphasizing the need for standardized methods to validate the quality and reliability of generated synthetic images.

## Conclusion

This systematic review identified 43 original studies published between 2019 and 2026, highlighting the rapid development of DMFR-specific Gen-AI networks. These studies mainly focused on improving image quality, particularly through spatial resolution enhancement and artifact reduction in CBCT, generating highly realistic synthetic radiographs, simulating post-treatment anatomical outcomes, reconstructing 3D structures from limited data, and automating interpretation and reporting. Although objective performance metrics were frequently reported, only about half of the studies included clinician-based validation, and most codes and models were not accessible for independent evaluation. Clinically, Gen-AI models demonstrated great potential to improve image quality and support more efficient diagnosis and reporting. However, their integration into practice is limited by data quality issues, insufficient clinical validation, and ethico-legal concerns. In education, Gen-AI might expand learning opportunities by providing diverse synthetic datasets, but their educational value depends on the fidelity and robust validation of generated images. For research, Gen-AI could facilitate data augmentation and help address data scarcity, particularly for rare diseases whereas challenges remain regarding dataset representativeness and reproducibility. Future studies should focus on clinical validation, greater transparency and accessibility, and thorough evaluation of cost-effectiveness in diagnostic workflows to ensure the safe and effective implementation of DMFR-specific Gen-AI.

## Author contributions

Kuo Feng Hung (Conceptualization, Methodology, Study screening, Data collection, Writing-original draft preparation), Feng Wang (Conceptualization, Methodology, Writing-review and editing), Ming-Yi Lu (Conceptualization, Writing-review and editing), Xieqi Shi (Conceptualization, Writing-review and editing), Michael M. Bornstein (Conceptualization, Writing-review and editing), and Qi Yong H. Ai (Conceptualization, Methodology, Study screening, Data collection, Writing-original draft preparation). All authors have read and agreed to the published version of the manuscript.

## Supplementary Material

twag024_Supplementary_Data

## Data Availability

Not applicable.
