# Supplementary material for: Current evidence of generative artificial intelligence specifically developed for dental and maxillofacial radiology: a systematic review
Source: Dentomaxillofac Radiol. 2026 May 21;55(6):538–67. doi: 10.1093/dmfr/twag024 (PMC13519356; doi:10.1093/dmfr/twag024)
Supplement: Supplementary file 1 [file twag024_supplementary_data.docx]

**Supplementary materials**

**Supplementary Table S1.** exhibits the specific search strategies used for all databases

| **Search on 10th December 2025** | |
| --- | --- |
| **Search strategy** | **Group 1 (Generative AI Model Terms, combined with ORs):** Terms relating to generative artificial intelligence models.  **Group 2 (Oral/Dental/Maxillofacial Imaging Terms, combined with ORs):** Terms identifying oral, dental, and maxillofacial radiology and imaging.  **AND:** The two groups are combined with AND, so only articles containing at least one term from each group are retrieved. |
| **Search Query** | |
| PubMed  (Record number = 363) | (("generative artificial intelligence"[All Fields] OR "generative AI"[All Fields] OR "gen AI"[All Fields] OR "large language model"[All Fields] OR "LLM"[All Fields] OR "transformer"[All Fields] OR "GAN"[All Fields] OR "diffusion model"[All Fields] OR "stable diffusion"[All Fields] OR "generative adversarial network"[All Fields] OR "variational autoencoder"[All Fields] OR "autoregressive model"[All Fields] OR "deep generative model"[All Fields] OR "image synthesis"[All Fields] OR "synthetic image"[All Fields] OR "image-to-image"[All Fields] OR "image-to-text"[All Fields] OR "text-to-image"[All Fields]))  **AND**  ("oral imaging"[All Fields] OR "oral radiology"[All Fields] OR "dental radiology"[All Fields] OR "dental imaging"[All Fields] OR "dental radiography"[All Fields] OR "maxillofacial imaging"[All Fields] OR "dentomaxillofacial radiology"[All Fields] OR "DMFR"[All Fields] OR "OMFR"[All Fields] OR "cone-beam computed tomography"[All Fields] OR "CBCT"[All Fields] OR "periapical radiography"[All Fields] OR "panoramic radiography"[All Fields] OR "OPG"[All Fields] OR "cephalometric radiography"[All Fields] OR "dental x-ray"[All Fields] OR "Radiology, Dental"[MeSH] OR "Dental Radiography"[MeSH] OR "Tomography, Cone-Beam Computed"[MeSH] OR "Maxillofacial Region"[MeSH])) |
| [Medline (via Ovid)](https://eproxy.lib.hku.hk/login?url=http://gateway.ovid.com/ovidweb.cgi?T=JS&MODE=ovid&PAGE=main&NEWS=n&DBC=y&D=mesz)  (Record number = 208) | ("generative artificial intelligence".mp. or  "generative AI".mp. or  "gen AI".mp. or  "large language model".mp. or  LLM.mp. or  transformer.mp. or  GAN.mp. or  "diffusion model".mp. or  "stable diffusion".mp. or  "generative adversarial network".mp. or  "variational autoencoder".mp. or  "autoregressive model".mp. or  "deep generative model".mp. or  "image synthesis".mp. or  "synthetic image".mp. or  "image-to-image".mp. or  "image-to-text".mp. or  "text-to-image".mp.)  and  ("oral imaging".mp. or  "oral radiology".mp. or  "dental radiology".mp. or  "dental imaging".mp. or  "dental radiography".mp. or  "maxillofacial imaging".mp. or  "dentomaxillofacial radiology".mp. or  DMFR.mp. or  OMFR.mp. or  "cone-beam computed tomography".mp. or  CBCT.mp. or  "periapical radiography".mp. or  "panoramic radiography".mp. or  OPG.mp. or  "cephalometric radiography".mp. or  "dental x-ray".mp. or  Radiology, Dental.sh. or  Dental Radiography.sh. or  Tomography, Cone-Beam Computed.sh. or  Maxillofacial Region.sh.) |
| [Embase](https://eproxy.lib.hku.hk/login?url=http://gateway.ovid.com/ovidweb.cgi?T=JS&MODE=ovid&PAGE=main&NEWS=n&DBC=y&D=mesz) (via Ovid)  (Record number = 524) | ("generative artificial intelligence".mp. or  "generative AI".mp. or  "gen AI".mp. or  "large language model".mp. or  LLM.mp. or  transformer.mp. or  GAN.mp. or  "diffusion model".mp. or  "stable diffusion".mp. or  "generative adversarial network".mp. or  "variational autoencoder".mp. or  "autoregressive model".mp. or  "deep generative model".mp. or  "image synthesis".mp. or  "synthetic image".mp. or  "image-to-image".mp. or  "image-to-text".mp. or  "text-to-image".mp.)  and  ("oral imaging".mp. or  "oral radiology".mp. or  "dental radiology".mp. or  "dental imaging".mp. or  "dental radiography".mp. or  "maxillofacial imaging".mp. or  "dentomaxillofacial radiology".mp. or  DMFR.mp. or  OMFR.mp. or  "cone-beam computed tomography".mp. or  CBCT.mp. or  "periapical radiography".mp. or  "panoramic radiography".mp. or  OPG.mp. or  "cephalometric radiography".mp. or  "dental x-ray".mp. or  Radiology, Dental.sh. or  Dental Radiography.sh. or  Tomography, Cone-Beam Computed.sh. or  Maxillofacial Region.sh.) |
| Web of Science  (Record number = 353) | TS=("generative artificial intelligence" OR "generative AI" OR "gen AI" OR "large language model" OR "LLM" OR "transformer" OR "GAN" OR "diffusion model" OR "stable diffusion" OR "generative adversarial network" OR "variational autoencoder" OR "autoregressive model" OR "deep generative model" OR "image synthesis" OR "synthetic image" OR "image-to-image" OR "image-to-text" OR "text-to-image")  **AND**  TS=("oral imaging" OR "oral radiology" OR "dental radiology" OR "dental imaging" OR "dental radiography" OR "maxillofacial imaging" OR "dentomaxillofacial radiology" OR "DMFR" OR "OMFR" OR "cone-beam computed tomography" OR "CBCT" OR "periapical radiography" OR "panoramic radiography" OR "OPG" OR "cephalometric radiography" OR "dental x-ray") |
| Scopus  (Record number = 612) | (TITLE-ABS-KEY("generative artificial intelligence" OR "generative AI" OR "gen AI" OR "large language model" OR "LLM" OR "transformer" OR "GAN" OR "diffusion model" OR "stable diffusion" OR "generative adversarial network" OR "variational autoencoder" OR "autoregressive model" OR "deep generative model" OR "image synthesis" OR "synthetic image" OR "image-to-image" OR "image-to-text" OR "text-to-image")) AND (TITLE-ABS-KEY("oral imaging" OR "oral radiology" OR "dental radiology" OR "dental imaging" OR "dental radiography" OR "maxillofacial imaging" OR "dentomaxillofacial radiology" OR "DMFR" OR "OMFR" OR "cone-beam computed tomography" OR "CBCT" OR "periapical radiography" OR "panoramic radiography" OR "OPG" OR "cephalometric radiography" OR "dental x-ray")) |

**Supplementary Table S2.** provides a summary of the reasons for excluding studies after full-text assessment.

| **Authors (Year)** | **Reason for exclusion** |
| --- | --- |
| Ren et al. (2016) | Use of hardware- and algorithm-based scatter correction method but not Gen-AI to remove the scatter artifacts in CBCT |
| Liang et al. (2017) | Use of rule-based algorithms but not Gen-AI to reduce artifacts from CBCT scans |
| Qiu et al. (2018) | Full-text not accessible |
| Yun et al. (2019) | Use of rule-based algorithms but not Gen-AI to improve the contrast of panoramic radiographs |
| Loi et al. (2020) | Use of rule-based algorithms but not Gen-AI for deformable image registration of CT and CBCT scans |
| Huang et al. (2021) | Full-text not accessible |
| Fu et al. (2024) | Use of Gen AI to enhance CBCT scans for image-guided thoracic cancer radiation therapy rather than DMFR tasks |
| Yuan et al. (2024) | Use of Gen AI to enhance CBCT scans for image-guided adaptive radiotherapy in nasopharyngeal carcinoma rather than for DMFR tasks |
| Fan et al. (2024) | Use of Gen AI to reduce metal artifacts from metallic appliances in fractured long bones after orthopaedic surgery rather than for DMFR tasks |
| Peng et al. (2024) | Use of Gen AI to enhance CBCT scans for image-guided radiation therapy in head and neck cancer rather than for DMFR tasks |
| Dong et al. (2025) | Use of Gen AI to enhance CBCT scans for image-guided radiation therapy in non-small cell lung cancer rather than DMFR tasks |
| Guo et al. (2025) | Use of Gen AI to reduce metal artifacts in body scans rather than for DMFR tasks |
| Zhang et al. (2025) | Use of Gen AI to enhance body scans for image-guided radiation therapy rather than for DMFR tasks |
| Deng et al. (2025) | Use of Gen AI to enhance body scans for image-guided radiation therapy rather than for DMFR tasks |
| Hu et al. (2026) | Use of Gen AI to enhance CBCT scans for image-guided radiation therapy in head and neck cancer rather than for DMFR tasks |

**Supplementary Table S3.** Risk-of-bias assessment scores of the included studies according to customized criteria adapted from the CLAIM evaluating four major domains: “Dataset”, “Gen AI network”, “Model performance evaluation”, and “Model transparency”. Each domain was assessed for concerns regarding the risk of bias in methodological quality and rated on a 3-point scale reflecting these concerns as low, moderate, or high.

|  | **Dataset** | **Gen AI network** | **Model performance evaluation** | **Model transparency** |
| --- | --- | --- | --- | --- |
| **Image quality (spatial-resolution)** | | | | |
| Moran et al. (2021) [16] | Low risk | Low risk | Low risk | High risk due to the lack of openly accessible code |
| Mohammad-Rahimi et al. (2023) [17] | Low risk | Low risk | Low risk | High risk due to the lack of openly accessible code |
| Celik et al. (2024) [18] | Low risk | Low risk | Moderate risk due to the lack of subjective assessment | High risk due to the lack of openly accessible code |
| Chen et al. (2024) [19] | Low risk | Low risk | Moderate risk due to the lack of subjective assessment | High risk due to the lack of openly accessible code |
| Chen et al. (2026) [20] | Low risk | Low risk | Low risk | High risk due to the lack of openly accessible code |
| Sabry et al. (2025) [21] | Low risk | Low risk | Low risk | High risk due to the lack of openly accessible code |
| **Image quality (improvement of image geometry and projection)** | | | | |
| Kim et al. (2023) [22] | Low risk | Low risk | Low risk | High risk due to the lack of openly accessible code |
| Kolukisa et al. (2025) [23] | Low risk | Low risk | Low risk | High risk due to the lack of openly accessible code |
| Kim et al. (2025) [24] | Low risk | Low risk | Low risk | High risk due to the lack of openly accessible code |
| **Image quality (noise reduction)** | | | | |
| Hegazy et al. (2020) [25] | Low risk | Low risk | Moderate risk due to the lack of subjective assessment | High risk due to the lack of openly accessible code |
| Zhang et al. (2026) [26] | Low risk | Low risk | Low risk | Low risk |
| **Image quality (quantitative accuracy of voxel values)** | | | | |
| Yong et al. (2021) [27] | Low risk | Low risk | Moderate risk due to the lack of subjective assessment | High risk due to the lack of openly accessible code |
| Kang et al. (2023) [28] | Low risk | Low risk | Moderate risk due to the lack of subjective assessment | High risk due to the lack of openly accessible code |
| **Image quality (artifact reduction)** | | | | |
| Wang et al. (2019) [29] | Moderate risk due to insufficient details provided regarding the source of images in the dataset | Low risk | Moderate risk due to the lack of subjective assessment | High risk due to the lack of openly accessible code |
| Sha et al. (2024) [30] | Low risk | Low risk | Moderate risk due to the lack of subjective assessment | Low risk |
| Choi et al. (2025) [31] | Moderate risk due to insufficient details provided regarding the source of images in the dataset | Low risk | Moderate risk due to the lack of subjective assessment | High risk due to the lack of openly accessible code |
| Zhang et al. (2025) [32] | Low risk | Low risk | Low risk | High risk due to the lack of openly accessible code |
| Wang et al. (2025) [33] | Moderate risk due to insufficient details provided regarding the source of images in the dataset | Low risk | Moderate risk due to the lack of subjective assessment | High risk due to the lack of openly accessible code |
| Wang et al. (2026) [34] | Moderate risk due to insufficient details provided regarding the source of images in the dataset | Low risk | Low risk | High risk due to the lack of openly accessible code |
| Oliveira et al. (2025) [35] | Low risk | Low risk | Low risk | High risk due to the lack of openly accessible code |
| Candemil et al. (2025) [36] | Low risk | Low risk | Low risk | High risk due to the lack of openly accessible code |
| Ko et al. (2020) [37] | Low risk | Low risk | Moderate risk due to the lack of subjective assessment | Low risk |
| Park et al. (2022) [38] | Low risk | Low risk | Moderate risk due to the lack of subjective assessment | High risk due to the lack of openly accessible code |
| Park et al. (2025) [39] | Low risk | Low risk | Moderate risk due to the lack of subjective assessment | High risk due to the lack of openly accessible code |
| **Image simulation (simulation without corresponding resources)** | | | | |
| Kirkwood et al. (2025) [40] | Low risk | Low risk | Low risk | High risk due to the lack of openly accessible code |
| Kim et al. (2021) [41] | Low risk | Low risk | Low risk | High risk due to the lack of openly accessible code |
| Huang et al. (2021) [42] | Low risk | Low risk | Moderate risk due to the lack of subjective assessment | High risk due to the lack of openly accessible code |
| Schoenhof et al. (2024) [43] | Low risk | Low risk | Moderate risk due to the lack of objective assessment | High risk due to the lack of openly accessible code |
| Pedersen et al. (2025) [44] | Low risk | Low risk | Low risk | Low risk |
| Yu et al. (2025) [45] | Low risk | Low risk | Moderate risk due to the lack of subjective assessment | High risk due to the lack of openly accessible code |
| Waqas et al. (2025) [46] | Low risk | Low risk | Low risk | High risk due to the lack of openly accessible code |
| Fukuda et al. (2024a) [47] | Low risk | Low risk | Low risk | Low risk |
| **Image simulation (image-to-image translation)** | | | | |
| Ryu et al. (2024) [48] | Low risk | Low risk | Moderate risk due to the lack of subjective assessment | High risk due to the lack of openly accessible code |
| Fukuda et al. (2024b) [49] | Low risk | Low risk | Low risk | High risk due to the lack of openly accessible code |
| Fukuda et al. (2024c) [50] | Low risk | Low risk | Low risk | High risk due to the lack of openly accessible code |
| Ha et al. (2025) [51] | Low risk | Low risk | Low risk | High risk due to the lack of openly accessible code |
| **Image simulation (post-treatment prediction simulation)** | | | | |
| Park et al. (2022) [52] | Low risk | Low risk | Low risk | High risk due to the lack of openly accessible code |
| Liang et al. (2020) [53] | Low risk | Low risk | Moderate risk due to the lack of objective assessment | Low risk |
| **3D *r*econstruction** | | | | |
| Mao et al. (2024) [54] | Low risk | Low risk | Moderate risk due to the lack of subjective assessment | High risk due to the lack of openly accessible code |
| Minhas et al. (2024) [55] | Low risk | Low risk | Moderate risk due to the lack of subjective assessment | High risk due to the lack of openly accessible code |
| Parida et al. (2025) [56] | Low risk | Low risk | Moderate risk due to the lack of subjective assessment | High risk due to the lack of openly accessible code |
| **Automated interpretation and reporting** | | | | |
| Gao et al. (2025) [57] | Moderate risk due to insufficient details provided regarding the number of ONJ-negative patients | Low risk | Low risk | Low risk |
| Dasanayaka et al. (2025) [58] | Moderate risk due to insufficient details provided regarding the source of images in the dataset | Low risk | Low risk | Low risk |
